# Kaposi's Varicelliform Eruption: A Serious and Potentially Fatal Complication of Darier's Disease

**DOI:** 10.7759/cureus.79077

**Published:** 2025-02-16

**Authors:** Rita Rosa Domingos, Pedro Reboredo, Ana Sara Monteiro, Cristina Sousa

**Affiliations:** 1 Internal Medicine, Unidade Local de Saúde do Algarve - Hospital de Faro, Faro, PRT; 2 Internal Medicine, Centro Hospitalar Universitário do Algarve - Hospital de Faro, Faro, PRT

**Keywords:** acyclovir, darier's disease, herpesvirus, kaposi varicelliform eruption, staphylococcal infections

## Abstract

Darier's disease (DD) is an autosomal dominant genetic disorder characterized by skin barrier dysfunction and chronic skin inflammation predisposing patients to secondary infections. Kaposi's varicelliform eruption (KVE), a severe viral infection, most commonly caused by herpes simplex virus (HSV), represents a life-threatening complication in these patients. The authors present the case of a 48-year-old woman with DD, who developed a progressive painful vesiculopustular eruption accompanied by systemic symptoms, including fever, bilateral otalgia, and ocular pain. The initial skin lesions, confined to the scapular region, rapidly spread to the face, periorbital region, and external ears. Clinical examination revealed vesiculopustular lesions, conjunctival hyperemia, and ear involvement. Laboratory testing confirmed HSV-1/2 immunoglobulin M (IgM) positivity, and blood cultures grew *Staphylococcus aureus*, indicating bacterial superinfection. The patient was treated with intravenous acyclovir and flucloxacillin, leading to significant clinical improvement. Clinicians should maintain a high index of suspicion for KVE in patients with DD presenting with vesicular rashes, particularly when systemic symptoms or rapid progression is observed. This case underscores the importance of early recognition of KVE in DD patients to facilitate timely antiviral and antibacterial treatment, reducing the risk of severe complications such as vision loss and permanent auditory impairment.

## Introduction

Darier's disease (DD) (also known as keratosis follicularis or dyskeratosis follicularis) is a rare autosomal dominant genetic disorder, with an estimated prevalence of 1:100,000 [1]. It typically presents during adolescence, emerging in the first or second decade of life. It is characterized by persistent yellowish-brown keratotic papules and plaques, predominantly affecting seborrheic and flexural regions such as the neck, chest, and groin. There is great variability in the extent of involvement ranging from only nail changes to generalized disease [2]. The condition is lifelong, following a chronic, relapsing course, with flare-ups triggered by trauma, elevated temperatures, perspiration, and ultraviolet radiation exposure [3]. As a result, the patients may have chronic pruritus, dysesthesia, and malodor of the skin, causing social and psychological distress.

This condition arises from a mutation in the ATP2A2 gene, which encodes the sarco-/endoplasmic reticulum calcium ATPase isoform 2 (SERCA2) protein. Mutations in SERCA2 manifest in keratinocytes, leading to dysregulated intracellular calcium signaling, impaired cell-to-cell adhesion, and dyskeratosis. Dyskeratosis refers to an abnormal premature keratinization in lower epidermal layers, disrupting normal differentiation, weakening skin integrity, and leading to hyperkeratotic papules. This facilitates colonization by opportunistic bacteria (*Staphylococcus aureus*, *Streptococcus* spp.) and overgrowth of fungal and viral pathogens (*Candida albicans*, herpes simplex virus (HSV)) [1,3,4].

Mutations in the ATP2A2 gene are also linked to the inflammatory dermal infiltrate of Th17 cells, leading to chronic skin inflammation [1,5].

Due to the keratinocyte dysfunction and the inflammatory dermal infiltrate, patients with DD exhibit heightened susceptibility to bacterial, fungal, and viral infections, exacerbated by keratinocyte debris accumulation [3].

Kaposi's varicelliform eruption (KVE) or eczema herpeticum is a rare and potentially fatal viral infection that is caused mainly by the reactivation of HSV, predominantly type 1, affecting individuals with pre-existing dermatological conditions, including DD [6,7].

Epidemiological data on KVE in patients with DD remain limited due to the rarity of the disease. These patients often experience significant and rapid clinical deterioration, necessitating hospitalization [6].

The cutaneous manifestations of KVE include painful vesiculopustular lesions, on an erythematous base. Lesions appear as monomorphic vesicles, pustules, or "punched-out" erosions with hemorrhagic or necrotic crusts, spreading rapidly across affected regions. Lesions can coalesce into larger ulcerative areas, increasing the risk of secondary bacterial infection (commonly *Staphylococcus aureus* or *Streptococcus pyogenes*). Lesions may heal with post-inflammatory hyperpigmentation or atrophic scarring in severe cases [8,9]. If periocular skin is involved, ophthalmologic complications may occur, such as conjunctivitis, periorbital edema, and HSV keratitis (corneal infection and inflammation), which may progress to corneal ulceration and blindness if untreated [10]. Although uncommon, HSV keratitis is a major cause of blindness worldwide [11-13]. Systemic manifestations of this disease include high fever, fatigue, and regional lymph node enlargement.

The diagnosis of KVE is mainly clinical: acute onset of monomorphic vesiculopustular lesions, predilection for areas with pre-existing dermatologic conditions, severe pain, systemic symptoms, rapid progression, and high risk of secondary bacterial infection. HSV diagnosis can be confirmed by direct HSV detection (polymerase chain reaction (PCR) or viral culture), serology (HSV-1/2 immunoglobulin M/immunoglobulin G (IgM/IgG)), or skin biopsy [14].

If recognized and treated early, lesions typically heal within 2-6 weeks. Delayed treatment increases the risk of scarring, ocular damage, systemic dissemination, and secondary bacterial infections. Therefore, KVE is a dermatologic emergency that necessitates the immediate initiation of antiviral therapy, even in the absence of diagnostic confirmation [6-9]. Although formal treatment guidelines for KVE are lacking, expert consensus supports acyclovir as the preferred first-line therapy [15,16]. Due to its modest oral bioavailability, intravenous administration of acyclovir is recommended in severe cases [17].

Given the high morbidity and risk of secondary bacterial sepsis, it is critical to maintain a high index of suspicion of KVE in DD patients presenting with new ulcerations, widespread erosions, or fever. Early diagnosis and immediate antiviral therapy are paramount to mitigating complications and mortality [8].

## Case presentation

We present the case of a 48-year-old woman with a longstanding history of DD, medicated with acitretin, a systemic retinoid, 10 mg daily (maintenance dose). The diagnosis of DD was made when the patient was 20 years old, compatible with the epidemiology of the disease. The patient's mother and grandfather also have this disease. As such, the suspicion for DD in this patient was high.

She presented with new painful, non-pruritic vesiculopustular lesions, accompanied by ocular pain, bilateral otalgia, and fever.

She reports that the initial eruption appeared in the scapular region, associated with severe pain, and subsequently spread to the neck. She sought medical attention at a local clinic, was diagnosed with a bacterial infection of the skin, and was prescribed oral amoxicillin.

Despite completing the prescribed antibiotic, over the following week, her cutaneous lesions progressively worsened, extending to the face, periorbital region, and external ears, leading her to come to the emergency department of the hospital.

Physical examination revealed a blood pressure of 110/84 mmHg and a heart rate of 101 beats per minute (bpm). Maximal temperature measured 40.5°C (auricular).

**Figure 1:**
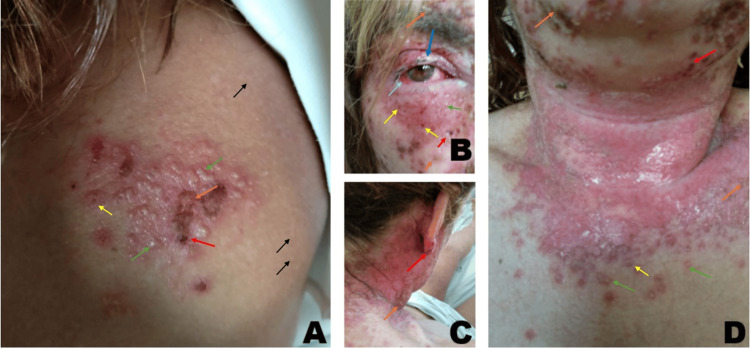
Kaposi's varicelliform eruption involving the right scapula (A), face and eyes (B), and scalp and cervical region (C, D). (A, C, D) Green arrows: vesiculopustular lesions; yellow arrows: punched-out ulcers; red arrows: hemorrhagic crusts; orange arrows: golden-brown patches; black arrows: small, firm, greasy, skin-colored to brown papules with a rough appearance, characteristic of Darier's disease. (B) Light blue arrow: conjunctival hyperemia; green arrow: periorbital vesicles; dark blue arrow: eyelid edema

The patient exhibited vesiculopustular lesions (green arrows) involving the right scapula (Figure 1A), face and eyes (Figure 1B), and scalp and cervical region (Figure 1C, 1D). They were interspersed with punched-out ulcers (yellow arrows) and hemorrhagic crusts (red arrows). The lesions had erythematous borders, with a large ulcerative area in the cervical region. This rouses the clinical suspicion of KVE.

Golden-brown patches (orange arrows) were also visible, increasing the clinical suspicion of secondary impetiginization (*Staphylococcus* superinfection).

Similar lesions were observed in the external and middle ear. Ophthalmologic assessment revealed conjunctival hyperemia (Figure 1B, light blue arrow), periorbital vesicles (Figure 1B, green arrow), and eyelid edema (Figure 1B, dark blue arrow), consistent with HSV involvement. The cornea remained clear, with no evidence of inflammation. Fundoscopic evaluation revealed a well-defined, well-perfused optic disc, a normal macula, and retinal vessels without pathological alterations, with no signs of retinal necrosis.

The remaining skin exhibited small, firm, greasy, skin-colored to brown papules with a rough appearance, characteristic of DD (black arrows). The patient exhibited extensive disease, with involvement of the face and periocular areas. She was therefore admitted to an internal medicine ward. Blood work and two sets of blood cultures were drawn (Table 1).

**Table 1:** Laboratory results day 1 versus day 10. CRP: C-reactive protein; HIV: human immunodeficiency virus; MSSA: methicillin-susceptible *Staphylococcus aureus*; HSV: herpes simplex virus; IgM: immunoglobulin M; IgG: immunoglobulin G

Parameter	Day 1	Day 10	Reference values
Hemoglobin	154 g/L	128 g/L	120-150 g/L
Leukocytes	3.5 × 10⁹/L	4.3 × 10⁹/L	4-10 × 10^9^/L
Platelets	267 × 10⁹/L	390 × 10⁹/L	150-400 × 10^9^/L
Bilirubin	0.3 mg/dL	-	0.2-1.2 mg/dL
Blood urea nitrogen	17 mg/dL	7 mg/dL	7-18.7 mg/dL
Creatinine	0.8 mg/dL	0.6 mg/dL	0.6-1.1 mg/dL
CRP	86 mg/L	<3 mg/L	<5 mg/L
HIV	Negative	-	-
Blood cultures	MSSA positive	-	-
Serologies	HSV-1/2 IgM positive, IgG negative	-	-

Given the clinical suspicion of KVE complicated by bacterial superinfection, the patient was immediately started on intravenous acyclovir (600 mg every eight hours) and flucloxacillin (2 g every six hours).

Laboratory investigations confirmed HSV-1/2 IgM positivity (IgG negative). Blood cultures grew multi-sensitive *Staphylococcus aureus*. Given this finding, an echocardiography was performed, ruling out endocarditis.

**Figure 2:**
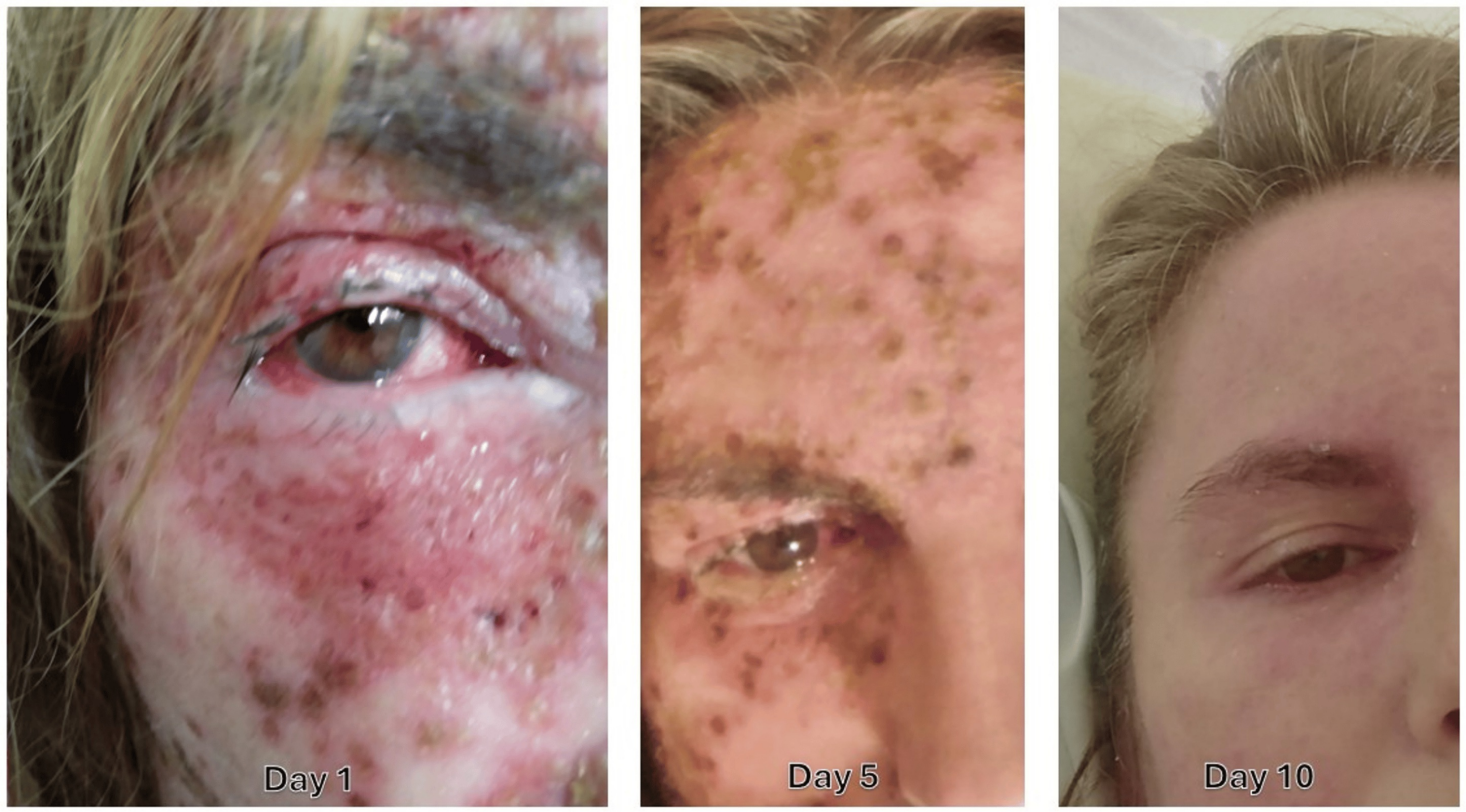
Follow-up of the patient's face lesions on days 1, 5, and 10.

The patient defervesced within 48 hours. Follow-up blood cultures were negative. She was discharged at day 10, with almost complete disappearance of lesions of KVE (Figure 2), and referred to her attending dermatologist, according to her preferences.

## Discussion

Due to the lack of clinical suspicion among healthcare professionals resulting from the rarity of the disease, KVE is often misdiagnosed as a bacterial infection, leading to delays in appropriate antiviral treatment.

In this case, the delayed initiation of acyclovir, commenced more than a week after symptom onset, allowed the infection to progress extensively, with high fever and widespread vesiculopustular lesions involving the face, eyes, and ears.

HSV is capable of inducing necrosis and ulceration, and delayed therapy may result in keratitis, corneal scarring, and blindness [9]. Fortunately, HSV keratitis was excluded in this case.

Scarring and post-inflammatory hyperpigmentation are significantly heightened with delayed treatment, which can impact the patient's long-term quality of life. At day 10, almost all the lesions had disappeared, although with some erythema remaining.

The development of *Staphylococcus aureus* bacteremia further complicated the clinical course, underscoring the susceptibility of DD patients to secondary bacterial infections. The presence of widespread erosions and ulcerations serves as an entry point for bacterial colonization, predisposing patients to systemic infections, including sepsis and endocarditis, excluded in this case. This highlights the importance of early antimicrobial intervention in suspected bacterial superinfection [1].

These findings reinforce the importance of considering both viral and bacterial co-infections in such cases. 

Therefore, early initiation of intravenous acyclovir is essential, even in the absence of immediate diagnostic confirmation [7]. 

## Conclusions

This case highlights the necessity for clinicians to maintain a high index of suspicion for KVE in patients with DD presenting with acute vesiculopustular eruptions, particularly in the presence of systemic symptoms.

The delayed initiation of antiviral therapy allowed for significant disease progression, involving the face, eyes, and ears, and was further complicated by systemic symptoms and bacterial superinfection. The ocular involvement posed a substantial risk of corneal scarring and potential permanent vision loss, while auricular extension heightened the risk of secondary infections and long-term auditory impairment. The confirmation of *Staphylococcus aureus* bacteremia further exacerbated disease severity and increased the risk of systemic dissemination. Given the previously stated, prompt recognition and early therapy are paramount.

This report reinforces the necessity of early recognition and initiation of systemic antiviral therapy in KVE, as a dermatologic emergency, alongside prompt antibacterial coverage, to mitigate complications, reduce hospitalization rates, and optimize patient outcomes.
